# Aerobic midgut microbiota of sand fly vectors of zoonotic visceral leishmaniasis from northern Iran, a step toward finding potential paratransgenic candidates

**DOI:** 10.1186/s13071-018-3273-y

**Published:** 2019-01-07

**Authors:** Fateh Karimian, Hassan Vatandoost, Yavar Rassi, Naseh Maleki-Ravasan, Mehdi Mohebali, Mohammad Hasan Shirazi, Mona Koosha, Nayyereh Choubdar, Mohammad Ali Oshaghi

**Affiliations:** 1 Department of Medical Entomology and Vector Control, School of Public Health, Tehran University of Medical Sciences (TUMS), Tehran, Iran; 2 Department of Parasitology, Pasteur Institute of Iran, Tehran, Iran; 3 Department of Medical Parasitology and Mycology, School of Public Health, Tehran University of Medical Sciences, Tehran, Iran; 4 Department of Pathobiology, School of Public Health, Tehran University of Medical Sciences, Tehran, Iran

**Keywords:** Sand fly, Microbiota, Paratransgenesis, Leishmaniasis, Iran

## Abstract

**Background:**

Leishmaniasis is caused by *Leishmania* parasites and is transmitted to humans through the bite of infected sand flies. Development of *Leishmania* to infective metacyclic promastigotes occurs within the sand fly gut where the gut microbiota influences development of the parasite. Paratransgenesis is a new control method in which symbiotic bacteria are isolated, transformed and reintroduced into the gut through their diet to express anti-parasitic molecules. In the present study, the midgut microbiota of three sand fly species from a steppe and a mountainous region of northern Iran, where zoonotic visceral leishmaniasis (ZVL) is endemic, was investigated.

**Methods:**

Briefly, adult female sand flies was collected during summer 2015 and, after dissection, the bacterial composition of the guts were analyzed using a culture-dependent method. Bacterial DNA from purified colonies was extracted to amplify the *16S* rRNA gene which was then sequenced.

**Results:**

Three ZVL sand fly vectors including *Phlebotomus major*, *P. kandelakii* and *P. halepensis* were found in the highlighted regions. In total, 39 distinct aerobic bacterial species were found in the sand fly midguts. The sand fly microbiota was dominated by Proteobacteria (56.4%) and Firmicutes (43.6%). Bacterial richness was significantly higher in the steppe region than in the mountainous region (32 *vs* 7 species). *Phlebotomus kandelakii*, the most important ZVL vector in the study area, had the highest bacterial richness among the three species. *Bacillus subtilis* and *Pantoea agglomerans* were isolated from the guts of the sand flies; these are already used for the paratransgenesis of sand flies and mosquitoes, respectively.

**Conclusions:**

The existence of *B. subtilis* and *P. agglomerans* in the ZVL vectors and other sand fly species studied so far suggests that these two bacterial species are potential candidates for paratransgenic approach to prevent ZVL transmission. Further research needs to test the possible relationship between the gut microbiome richness and the vector competence of the ZVL vectors.

## Background

Sand flies are the major vectors of various *Leishmania* spp., the etiological parasitic agents of leishmaniasis, a neglected tropical disease (NTD). Several clinical forms of leishmaniasis have been described including cutaneous, mucocutaneous and visceral (also known as kala-azar) forms [1, 2]. Approximately 350 million people are at risk of leishmaniasis, with nearly 12 million people in tropical, subtropical and Mediterranean regions affected by the disease. Visceral leishmaniasis (VL) is endemic in more than 65 countries and caused by various *Leishmania* species, *L. donovani* and *L. infantum* having a major incidence. The case-fatality rate of VL is about 10% of an estimated 200,000–400,000 cases annually [3]. In Iran, zoonotic visceral leshmaniasis (ZVL) is caused mainly by *L. infatum* and is endemic in the northwestern and southern regions with 100–300 new cases every year [4–6]. More than 30% of ZVL cases have been reported in northwestern areas. The disease is highly prevalent (> 90%) in children less than ten years-old where domestic dogs and other canines are reservoir hosts of the disease [5–7]. Several sand fly species, including *Phlebotomus perfiliewi transcaucasicus*, *P. kandelakii* and *P. tobbi* in northeastern and northwestern and *P. major* (*s.l.*) (*P. neglectus*), *P. keshishiani* and *P. alexandri* in southern areas of the country, were incriminated as possible vectors of ZVL [8–13]. The lack of an effective vaccine against leishmaniasis, narrow ranges of effectiveness and unfavorable side effects of available drugs, and development of drug resistance in the parasite highlight the need for novel approaches to control vector transmission of *L. infantum* [14].

Paratransgenesis is an alternative control strategy, where commensal or symbiont bacteria found in insect vector/s is engineered to inhibit pathogen transmission [15–17]. The principal and essential step in paratransgenesis is the identification of suitable bacterium/bacteria in the vector. The characteristics required for a candidate include being non-pathogenic to human and non-target animals, dominancy within the vector-associated microflora, cultivability in cell-free media, malleable to transformation with foreign DNA, and having a wide distribution [18]. Gut microbial communities, including bacterial species, have been investigated in various insects including blood sucking bugs [19], tsetse flies [20], mosquitoes [21–24] American cockroaches [25] and sand flies [26–28]. So far, researchers have nominated a few bacterial candidates for a paratransgenic approach to block *Leishmania* transmission in sand fly vectors including *Bacillus megaterium*, *Brevibacterium linens* and *Enterobacter cloacae* [16, 26, 28].

The digestive tract of *Phlebotomus* spp. is the main colonizing site of various microorganisms including bacteria. Sand flies acquire bacteria from food and the soil in which they breed at the larval stage and *via* polluted sugar meals derived from plant leaves and fruits or aphid honeydew at the adult stage [2]. The skin of sand fly hosts (e.g. mammals and reptiles) is another source of bacteria for female insects when ingesting blood meals. However, these blood meals are usually sterile [29]. Although the midgut microbiota of insects is apparently a function of the host, feeding behavior and environment factors, the effects of these factors on the composition and diversity of sand fly gut microbiota are commonly indefinite. Some of these factors include genetics and physicochemical parameters of insect, larval habitats and vertebrate hosts, climate, geographical features, and soil and plant attributes [30–35]. The aim of this study was to assess the composition of aerobic gut bacterial communities in the kala-azar vector *Phlebotomus* species from two different geographical regions (steppe and mountainous) in north of Iran as well as the community composition between various populations of an identical species. This information is important for the better understanding of symbiotic or commensal relationships between the bacteria and sand flies, mechanisms that determine gut microbiota composition and introduction of a potential candidate for a paratransgenesis approach against leishmaniasis in the study areas.

## Methods

### Study area

**Fig. 1:**
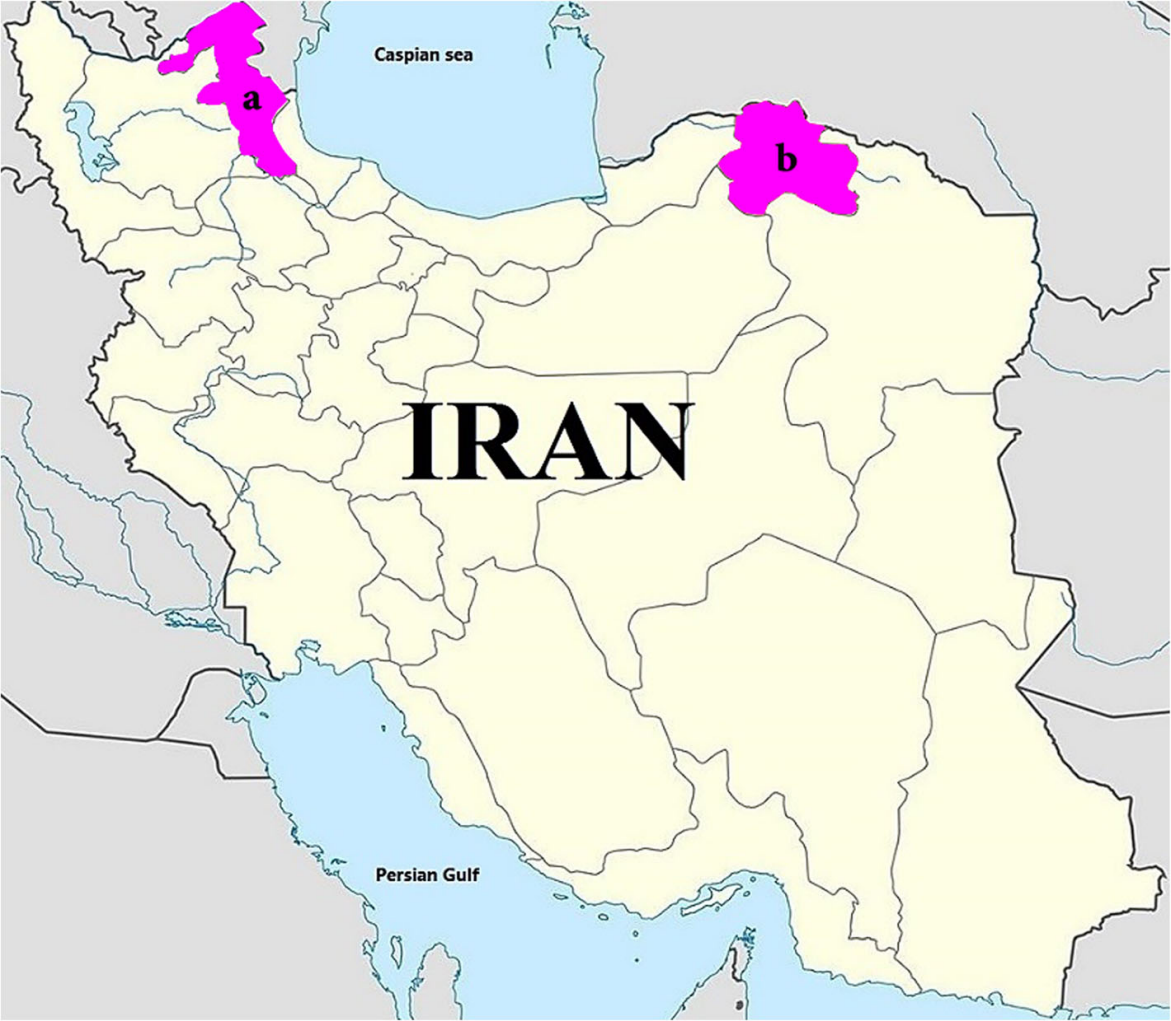
Map of two endemic visceral leishmaniasis (VL) foci in northwest and northeast of Iran where sand flies were collected. **a** Ardabil Province. **b** North Khorasan Province

The present study was carried out in two endemic foci of zoonotic visceral leishmaniasis in northeastern (North Khorasan Province) and northwestern (Ardabil Province) of Iran (Fig. 1). Up to 2012, a total of about 900 and 164 (44.6 and 8.2% of total cases in the country) VL cases have been reported from northwestern in northeastern regions of the country, respectively [4, 36]. North Khorasan Province (36°37'–38°17'N, 55°53'–58°20'E) is a mountainous region located in northeastern Iran, 1070 meters above sea level and with an area of more than 28,400 km^2^. The weather is hot (up to 32.4 °C) in summer and cold (below -3.4 °C) in winter, with an average annual temperature of 13.2 °C. This region is a desert and mountainous area and receives less than 250 mm rainfall annually. Ardabil Province (37°04'–39°65'N, 47°40'–48°71'E) is a steppe region located 1490 meters above sea level with an area of more than 17,800 square km2. The weather is hot (up to 40 °C) in summer and cold (below -20 °C) in winter with an average annual temperature of 9.5 °C. The warm season is short (mid-May to mid-September). The annual rainfall is approximately 325 mm and the climate is warm and temperate, considered to be a local steppe climate. Details of the climate data during sample collection are shown in Table 1.

**Table 1:** Climate data during the sample collection in the study areas

Location	Coordinates	Date	Season	T (°C)	Mean RH (%)	Rain (mm)	Mean wind speed (km/h)
Meshkinshahr	37°04'–39°65'N, 47°40'–48°71'E	2015	Late spring-early summer	7–31	59.31	0	18
Bojnurd	36°37'–38°17'N, 55°53'–58°20'E	2015	Late spring-early summer	12–39	47.2	0	9

*Abbreviations*: *T* temperature (range), *RH* relative humidity

### Sand fly midgut collection

To study the aerobic microbiota of the sand fly gut, wild sand flies were collected from the study areas using various methods including CDC light traps, car traps and aspirators during 2015. Live sand flies were immediately transferred to the Insect Molecular Biology Laboratory, Department of Medical Entomology and Vector Control, School of Public Health, Tehran University of Medical Science, Tehran, Iran, in cold chain condition.

### Isolation of bacteria

Female sand fly specimens (*n* = 117) were separated from male specimens (*n* = 324) and used for bacterial isolation. Female specimens were immediately micro-dissected individually under a microbiological safety cabinet on a sterile glass slide. First, specimens were surface sterilized using 70% ethanol for 3–5 min and then the gut of each specimen was micro-dissected and homogenized by shaking in a sterile tube containing glass beads. This was transferred to test screw tubes containing 5 ml of brain heart infusions (BHI) broth and incubated at 37 °C for 24–48 h in aerobic conditions. Cloudy test tubes were considered as positive specimens. The grown bacteria were serially diluted or streaked on BHI agar plates and subcultured several times on the agar plates in the same conditions to achieve individual purified colonies. The remainder of the dissected insect body was mounted on a slide for morphological identification. Following species identification, microbiota definition was carried out only for specimens corresponding to known VL vectors. Test tubes containing BHI broth were opened near the dissection area under sterile conditions during the micro-dissection processes. Individual colonies were selected and used for further molecular identification. The richness of each bacterial family was calculated based on the total number of *16S* sequences.

### *16S* rRNA gene amplification

DNA extraction from individual colonies was carried out using the phenol/chloroform DNA extraction method as previously described by Maleki-Ravasan et al. [26]. Nearly 1500 bp of the bacterial *16S* rRNA gene including the less variable V1-V2 and the highly variable V3-V5 regions were amplified using the universal primers 16suF (5'-GAG TTT GAT CCT GGC TCA G-3') and 16suR (5'-GTT ACC TTG TTA CGA CTT-3') [37]. Polymerase chain reaction (PCR) amplification was carried out using a Maxime PCR PreMix Kit (i-*Taq*) in 20 μl reaction mixtures containing 1 μl of each primer with 10 μM concentration and 1–2 μl (~0.1 μg) of extracted genomic DNA. BHI agar media and ddH_2_O were used as negative controls. The thermal cycler conditions were set as follows: an initial denaturation at 94 °C for 10 min, followed by 35 cycles of denaturation at 95 °C for 30 s, annealing at 57.5 °C for 40 s and extension at 72 °C for 30 s. Final extension was at 72 °C for 8 min. The PCR products were visualized on a 1% agarose gel containing ethidium bromide using a UV transilluminator. QIAquick PCR Purification Kit (Qiagen, Hilden, Germany) was used for purification of the PCR products before sequencing.

### *16S* rRNA gene sequencing and analysis

First, *16S* rRNA amplicons were amplified using each forward or reverse primer and then sequenced using the Sanger method (Macrogen, Seoul, S. Korea). To compare these sequences with those available in ribosomal databases, nine databases of prokaryotic *16S* rRNA gene were used, namely NCBI (nucleotide collection; http://blast.ncbi.nlm.nih.gov/Blast.cgi), EMBL (http://www.ebi.ac.uk/ena), RDP (http://rdp.cme.msu.edu/seqmatch/seqmatch_intro.jsp), EzTaxon-e (http://eztaxon-e.ezbiocloud.net), Greengenes (http://greengenes.lbl.gov/cgi-bin/nph-index.cgi), DDBJ (http://blast.ddbj.nig.ac.jp/?lang=en), leBIBI (http://umr5558-sud-str1.univ-lyon1.fr/lebibi/lebibi.cgi) and Blast2Tree (http://bioinfo.unice.fr/blast) [38, 39]. Sequence homology with available data was assessed based on the number and quality of nucleotides of the sequence reads using appropriate features of the data such as cultivable and/or non-cultivable and type and/or non-type specimens. The sequences were assigned at the species level based on either the most common nomenclature within the results of the nine databases or the highest similarity rate. Nucleotide homology > 95 and 98% were considered as lower thresholds at genus and species levels, respectively (https://rdp.cme.msu.edu). The partial *16S* rDNA consensus sequences obtained in this study were annotated in the GenBank database using the *16S* ribosomal RNA database (https://submit.ncbi.nlm.nih.gov/subs/genbank). Species assignation of the symbiont bacteria was verified by phylogenetic analysis using *16S* rRNA gene sequences based on the neighbor-joining algorithm of MEGA7 Software. Cytoscape Software (http://www.cytoscape.org), as a tool for visualizing complex networks among data, was used to visualize bacterial richness and shared bacteria in the three sand fly species through the network analysis [40]. Data, as CYS files containing vertices or nodes (representing symbiont bacteria and sand fly hosts) and edges (representing links), were submitted to Cytoscape software v.3.5.1. Bacterial and host nodes as well as geographical region links were colored for the better demonstration of their interaction. GraphPad Prism software v.5.00 for Windows (GraphPad, San Diego, USA) and Student’s t-test embedded in the software was used for graphical representation and statistical analysis, respectively.

## Results

In total, 1772 sand flies comprising 1565 (88.3%) *Phlebotomus* and 207 (11.7%) *Sergentomyia* specimens were collected from the study areas. The *Phlebotomus* species included ZVL vectors *P. major*, *P. kandelakii* and *P. halepensis*, and cutaneous leishmaniasis (CL) vectors *P. sergenti* and *P. papatasi*. Approximately 64.5% (*n* = 1142) of the specimens were male. After exclusion of *Sergentomyia* spp., the CL vectors, dead females and male specimens, 117 live female ZVL phlebotomine sand flies comprising 48 *P. kandelakii*, 51 *P. major* and 18 *P. halepensis* specimens were processed individually for their midgut bacterial composition. A total of 39 independent bacterial colonies or OTUs across six families was obtained from the midgut of the three field-collected sand fly species (Table 2).

**Table 2:** Details of bacterial richness in the midgut of three sand fly species from steppe (Bojnurd) and mountainous (Meshkinshahr) regions in northeastern Iran

Location	*P. major*			*P. kandelakii*			*P. halepensis*			Total		
	*n*	BS	BSPP	*n*	BS	BSPP	*n*	BS	BSPP	*n*	BS	BSPP
Bojnurd	33	18	12	36	16	12	18	8	8	87	42	32
Meshkinshahr	18	3	2	12	6	5	0	0	0	30	9	7
Total	51	21	14	48	22	17	18	8	8	117	51	39

*Abbreviations*: *n* number of sand flies, *BS* number of bacterial sequences, *BSPP* number of bacterial species

**Fig. 2:**
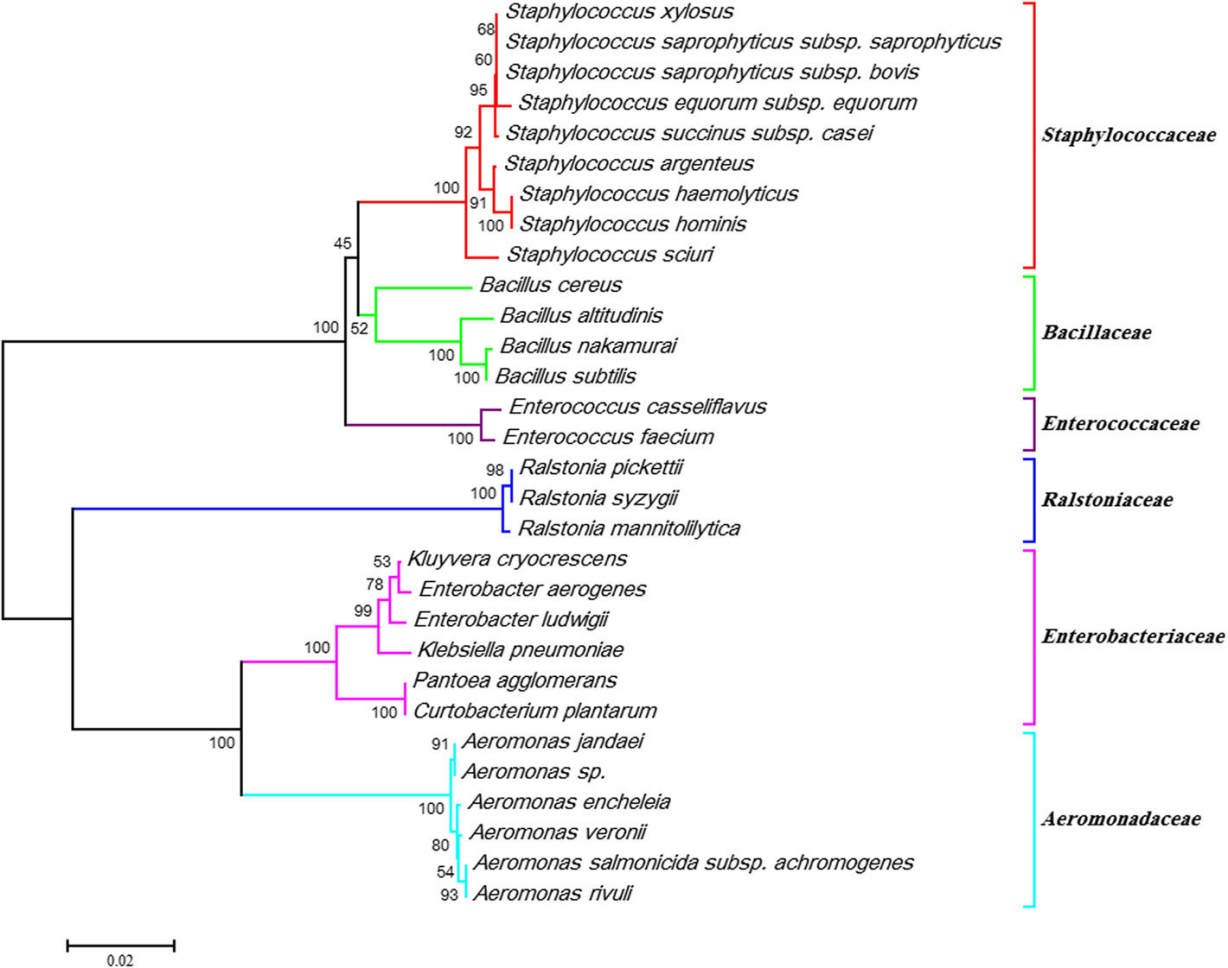
Phylogenetic analysis of gut microbiota isolated from *P. kandelakii* (k), *P. major* (m) and *P. halepensis* (h) sand flies verified by 980 bp of *16S* rRNA gene sequences. The sequences were aligned using Clustal Omega Software. The phylogenetic tree was constructed based on the neighbor-joining algorithm using MEGA7 software. Each bacterial family in the phylogenetic tree is represented by a separate colored line. Bootstrap values are shown at nodes. Scale of the genetic distance is shown underneath. *Abbreviations*: B, Bojnurd; M, Meshkinshahr

**Fig. 3:**
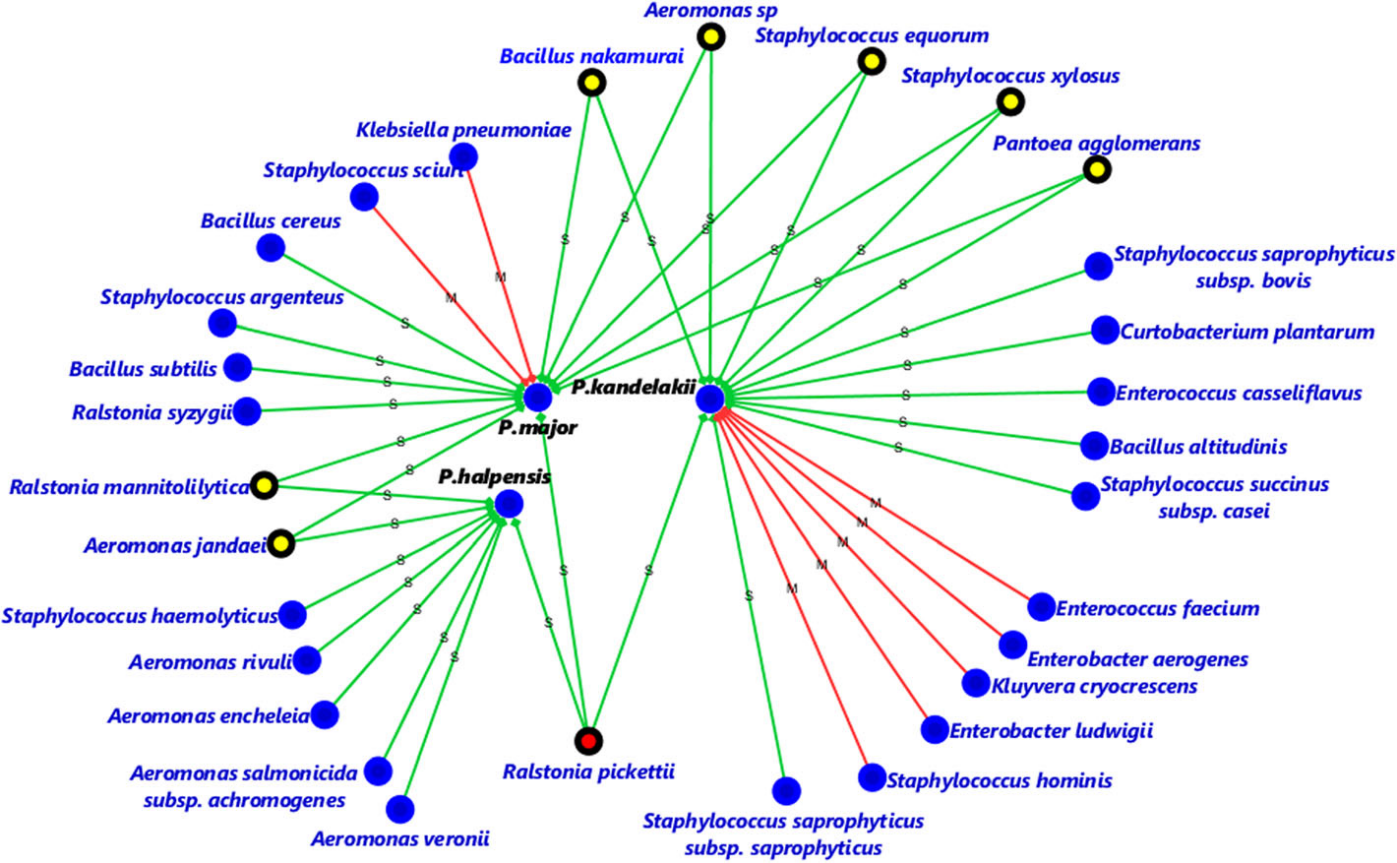
Network analysis showing the shared and non-shared bacteria species isolated from *P. kandelakii*, *P. major* and *P. halepensis* (red squares). The bacteria species observed in three, two and one hosts can be identified by red, yellow (surrounded by black line) and blue circles, respectively. Red and green lines represent mountainous and steppe regions, respectively

**Fig. 4:**
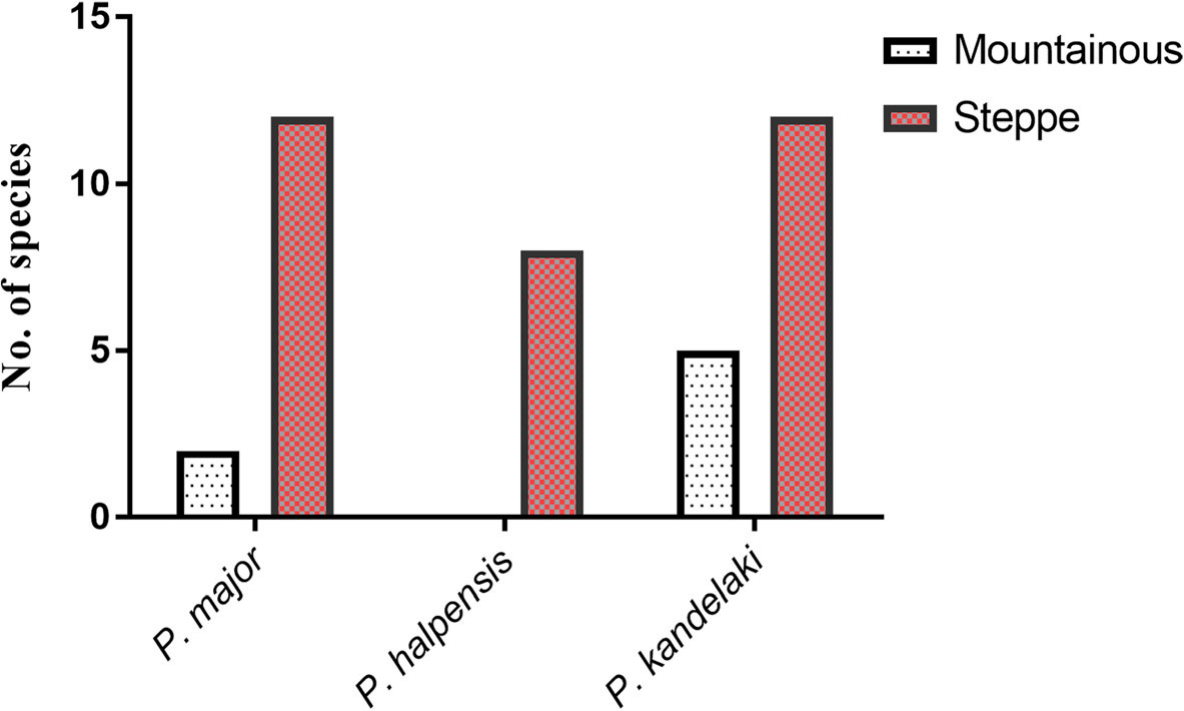
Number of bacteria species isolated from the midgut of three field collected sand fly species based on collection localities in two distinct areas in Iran

The phylogenetic relationships of the bacteria and their corresponding taxonomic status at family level, in addition to their host and collection sites are shown in Fig. 2. Members of *Bacillaceae*, *Ralstoniaceae* and *Aeromonadaceae* were reported only in the steppe region (Bojnurd). Furthermore, results showed that bacterial taxonomic richness of the steppe region sand flies was greater (32 *vs* 7 species) than that of moderate mountainous region. Tables 3 and 4 show details of 39 isolated bacteria from the midgut of *P. major*, *P. kandelakii* and *P. halepensis* from the study areas. *Phlebotomus kandelakii* midgut with 17 bacterial species or OTUs had the greatest bacterial richness among the three host species (Table 2 and Fig. 2). *Phlebotomus major* and *P. halepensis* harbored 14 and 8 bacterial species, respectively (Table 2 and Fig. 2). *Ralstonia pickettii* was the only shared bacteria found in all the three sand fly species in the steppe region (Fig. 3). There were six, three and one shared bacterial species between *P. major*-*P. kandelakii*, *P. major*-*P. halepensis* and *P. kandelakii*-*P. halepensis*, respectively, in the steppe region (Tables 3 and 4 and Figs. 3 and 4). No shared bacterium was reported between sand fly guts in the mountainous study area. In total, nearly 80% of the bacteria were observed in individual species only.

**Table 3:** Details of the bacteria isolated from the midgut of sand flies captured in the steppe region (Bojnurd) of northeastern Iran

Assigned bacterial spp.	Sand fly origin	Similarity %		Phylum	Family	GenBank ID
		EzTaxa	NCBI			
*Aeromonas* sp*.*	*P. major*	99.9	100	Proteobacteria	*Aeromonadaceae*	MF372627
	*P. kandelakii*	99.9	100	Proteobacteria	*Aeromonadaceae*	MF372629
*Staphylococcus equorum equorum*	*P. major*	100	100	Firmicutes	*Staphylococcaceae*	MF372605
	*P. kandelakii*	100	100	Firmicutes	*Staphylococcaceae*	MF372618
*Aeromonas jandaei*	*P. major*	99.7	99	Proteobacteria	*Aeromonadaceae*	MF372624
	*P. halepensis*	99.4	99	Proteobacteria	*Aeromonadaceae*	MF372620
*Staphylococcus xylosus*	*P. major*	100	100	Firmicutes	*Staphylococcaceae*	MF372602
	*P. kandelakii*	99.8	100	Firmicutes	*Staphylococcaceae*	MF372601
*Bacillus cereus*	*P. major*	100	100	Firmicutes	*Bacillaceae*	MF372604
*Bacillus nakamurai*	*P. major*	99.9	100	Firmicutes	*Bacillaceae*	MF372611
	*P. kandelakii*	99.9	100	Firmicutes	*Bacillaceae*	MF372607
*Bacillus subtilis*	*P. major*	99.9	99	Firmicutes	*Bacillaceae*	MF372612
*Pantoea agglomerans*	*P. major*	100	100	Proteobacteria	*Enterobacteriaceae*	MF372619
	*P. kandelakii*	100	100	Proteobacteria	*Enterobacteriaceae*	MF289172
*Ralstonia pickettii*	*P. major*	99.6	99	Proteobacteria	*Ralstoniaceae*	MF372616
	*P. kandelakii*	99.8	99	Proteobacteria	*Ralstoniaceae*	MF372617
	*P. halepensis*	99.9	99	Proteobacteria	*Ralstoniaceae*	MF372615
*Ralstonia mannitolilytica*	*P. major*	99.8	99	Proteobacteria	*Ralstoniaceae*	MF372614
	*P. halepensis*	99.6	99	Proteobacteria	*Ralstoniaceae*	MF372613
*Staphylococcus argenteus*	*P. major*	99.7	99	Firmicutes	*Staphylococcaceae*	MF372606
*Ralstonia syzygii*	*P. major*	99.7	99	Proteobacteria	*Ralstoniaceae*	MF372625
*Staphylococcus succinus casei*	*P. kandelakii*	99.9	99	Firmicutes	*Staphylococcaceae*	MF372608
*Staphylococcus saprophyticus bovis*	*P. kandelakii*	100	99	Firmicutes	*Staphylococcaceae*	MF372603
*Curtobacterium plantarum*	*P. kandelakii*	100	100	Proteobacteria	*Enterobacteriaceae*	MF372626
*Staphylococcus saprophyticus saprophyticus*	*P. andelakii*	100	100	Firmicutes	*Staphylococcaceae*	MF372628
*Enterococcus casseliflavus*	*P. kandelakii*	100	99	Firmicutes	*Enterococcaceae*	MF372609
*Bacillus altitudinis*	*P. kandelakii*	99.8	99	Firmicutes	*Bacillaceae*	MF372610
*Aeromonas veronii*	*P. halepensis*	99.8	*99*	Proteobacteria	*Aeromonadaceae*	MF372621
*Aeromonas salmonicida achromogenes*	*P. halepensis*	99.8	*99*	Proteobacteria	*Aeromonadaceae*	MF372622
*Staphylococcus haemolyticus*	*P. halepensis*	98.8	*99*	Firmicutes	*Staphylococcaceae*	MF372630
*Aeromonas encheleia*	*P. halepensis*	99.9	*100*	Proteobacteria	*Aeromonadaceae*	MF372631
*Aeromonas rivuli*	*P. halepensis*	99.9	*99*	Proteobacteria	*Aeromonadaceae*	MF372623

**Table 4:** Details of the bacteria isolated from the midgut of sand flies captured in the mountainous region (Meshkinshahr) of northwestern Iran

Assigned bacterial spp.	Sand fly origin	Similarity %		Phylum	Family	GenBank ID
		EzTaxa	NCBI			
*Kluyvera cryocrescens*	*P. kandelakii*	99.5	99	Proteobacteria	*Enterobacteriaceae*	MF372632
*Enterobacter aerogenes*	*P. kandelakii*	100	100	Proteobacteria	*Enterobacteriaceae*	MF372633
*Enterococcus faecium*	*P. kandelakii*	100	100	Proteobacteria	*Enterococcaceae*	MF372634
*Enterobacter ludwigii*	*P. kandelakii*	100	99	Proteobacteria	*Enterobacteriaceae*	MF372638
*Staphylococcus hominis*	*P. kandelakii*	99.9	100	Firmicutes	*Staphylococcaceae*	MF372635
*Klebsiella pneumoniae*	*P. major*	100	100	Proteobacteria	*Enterobacteriaceae*	MF372637
*Staphylococcus sciuri*	*P. major*	100	100	Firmicutes	*Staphylococcaceae*	MF372636

**Fig. 5:**
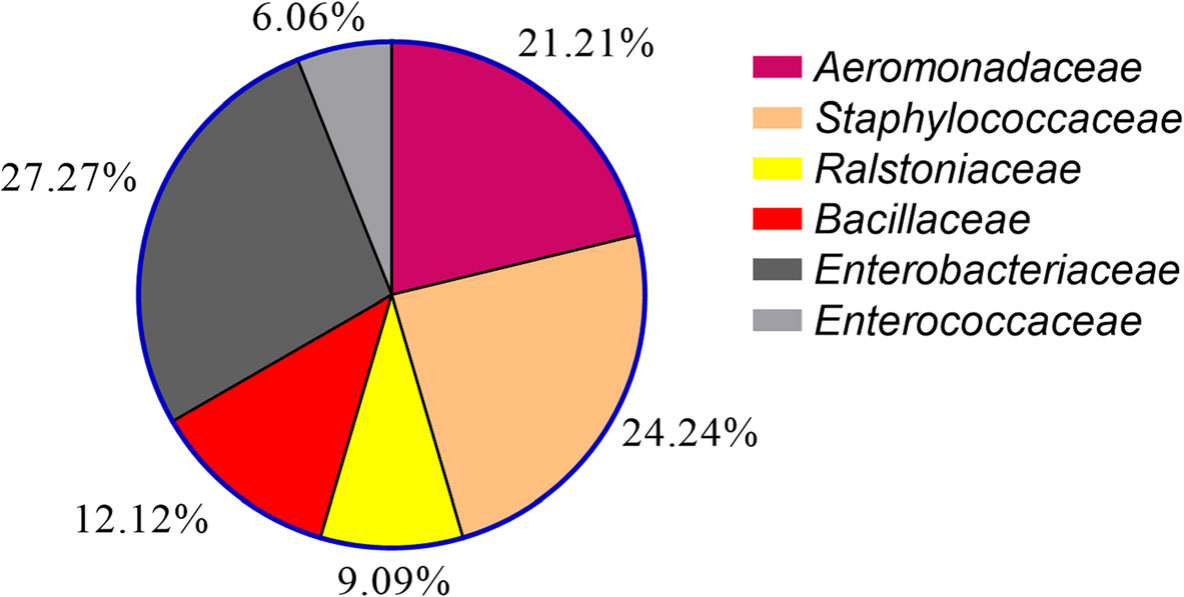
Relative frequency of bacterial families isolated from the midgut of *P. major*, *P. kandelakii* and *P. halepensis* in the study areas of Iran

The bacterial colonies isolated from sand fly midguts belonged to two phyla: Proteobacteria (56.41%) and Firmicutes (43.59%) across six families (Fig. 5). Based on oxygen demands, about 87.2 and 12.8% of the bacteria were assigned as aerobes and facultative anaerobes, respectively. The genus *Staphylococcus* (26.66%) and the family Enterobacteriaceae (27.27%) had the highest abundances in the sand fly species.

**Fig. 6:**
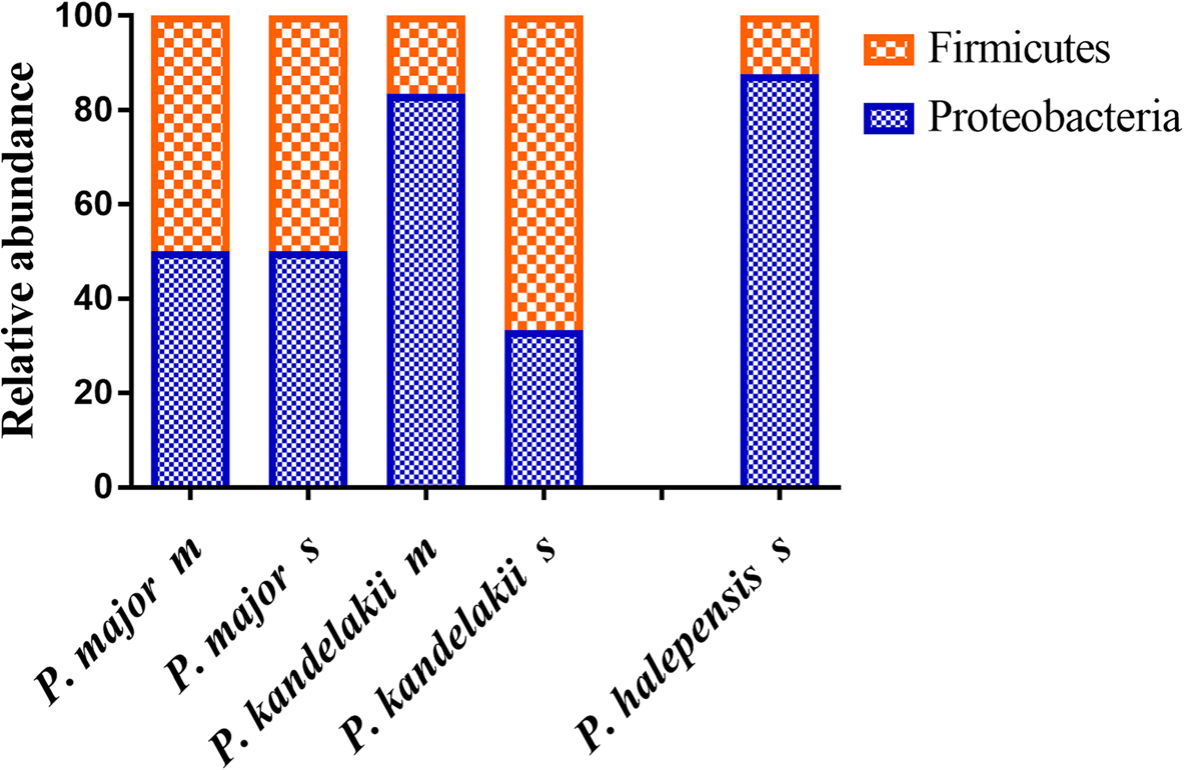
Relative frequency of aerobic Proteobacteria and Firmicutes grown in BHI media from the gut of sand fly species originating from steppe (s) and mountainous (m) regions in northern Iran

Except for *P. major*, there was notable differences in taxonomic composition between the bacterial microbiota among allopatric sand fly species (occurring in separate non-overlapping geographical areas), and sympatric (occurring within the same or overlapping geographical areas) sand flies (Fig. 6). In the steppe region, for *P. kandelakii*, the midgut bacterial composition was Proteobacteria (33.33%) and Firmicutes (66.67%), and for *P. halepensis* the midgut bacterial composition was Proteobacteria (87.50%) and Firmicutes (12.50%). In the mountainous region, for *P. kandelakii*, the midgut bacterial composition was Proteobacteria (83.34%) and Firmicutes (16.66 %) (Fig. 6). For *P.major*, the midgut bacterial composition was Proteobacteria (50%) and Firmicutes (50%) in both regions.

## Discussion

In the present study, gut aerobic microbiota of three different sand fly species was assessed using the culture dependent method and *16S* rRNA gene sequencing. Results showed that gut microbiota of these insects were dominated by Proteobacteria (56.41%) and Firmicutes (43.59%), in agreement with previous studies using either culture-dependent or independent methods (next-generation sequencing, NGS) showing that these two phyla were the predominant phyla in various groups of insects [26, 41]. Proteobacteria and Firmicutes with 57.4 and 21.7%, respectively, were predominant in 62 insect species. Yun et al. [42] showed that Proteobacteria with 62.1% and Firmicutes with 20.7% were the predominant phyla in 218 species belong to 21 insect taxonomic orders. Results of the present study showed that 80, 18 and 2% of the bacterial species were observed in a single, two and three sand fly species, respectively. This is similar to the results of previous studies demonstrating that most bacterial operational taxonomic units (OTUs) were restrained to a single environment [42, 43]. These species-specific compositions in gut microbiota of the sand flies can be explained by food sources of the insects at larval and adult stages and the environmental habitats in which they live. Food diet, comprising mostly sugar sources for adults and organic matters for larvae, play a major role in the microbiome community of sand flies. The lack of shared bacteria in the gut microbiota of the same sand fly species from two distinct regions (steppe and the mountainous) shows the great influence of environment on the community composition of insect gut microbiota. Interestingly, most bacteria in the mountainous region (five out of seven species) belonged to the family *Enterobacteriaceae*. In contrast, members of this family had the lowest frequency in the steppe region. This finding is supported by previous studies indicating a significant positive correlation between the gut microbiota and environmental conditions such as temperature and precipitation in various insect species [31, 32, 41]. Similar to other insects, sand flies are ectothermic insects and temperature affects their biology and fitness parameters such as longevity, survival and behavior [12, 44]. In the steppe region of this study, the climate is warmer than that of the mountainous region, which might support the additional richness of bacteria in sand fly guts of that region.

When the microbial diversity of the VL vectors of this study were compared with data of sand flies from the Old World [26, 45, 46], we found some similar bacteria such as the genera *Enterobacter* and *Staphylococcus* and more specifically *B. subtilis*. Additionally, a low number of bacteria found in this study were common to those found in the phlebotomine sand flies from the Old and New World, namely *B. subtilis*, *Staphylococcus* spp., *Enterobacter* spp. and *Klebsiella* spp.

Sand fly larvae breed in soil where they feed on decaying organic matter. Soil characteristics and microbial community may affect the bacterial composition of the gut of sand fly larvae. Furthermore, they might cause regional changes in the bacterial communities observed in this study. A strong correlation between insect microbiota and environmental habitat, including soil, has already been reported for some ground insects such as *P. argentipes* [28], *P. papatasi* [26] and *P. perniciosus* [35].

Soil characteristics such as chemistry, moisture, temperature, agricultural activity and plant and animal species, noticeably affect soil bacterial diversity [41]. Soil characteristics and agricultural practices in the steppe and mountainous areas of the present study were completely different; this might have affected the bacterial community structure in soil and resulted in the bacterial community variations. However, it is worth mentioning that only a few bacterial communities can pass transstadially from larval to adult stages [29].

Host physiological condition, gut morphology and food sources are important factors for insect microbial diversity [36, 42]. Although no detailed information on the similarity of gut morphology and physicochemical conditions (pH, oxygen availability, redox conditions and digestive enzyme) have been reported in *P. kandelakii*, *P. major* and *P. halepensis*, and remain still to be investigated, it seems that no significant differences exist between these characteristics in these close species. Therefore, diverse sources of bacteria might influence the gut microbiota of these three species with different habitats, behaviors and diets. Sand fly larvae are scavengers feeding on dead organic matter such as animal feces, bacteria, algae and fungi in sewage and organic sludge. In contrast, adult sand flies feed on flower nectar and plant sap, and adult female sand flies suck blood from humans, mammals and reptiles. Adult sand flies may acquire bacteria from plant sources and the skin of the hosts while sucking blood. Furthermore, bacteria may be transstadially transmitted from larval to adult midguts [16]. The microbiota of *P. kandelakii*, with 17 bacterial species, has the greatest richness among the three sand fly species. This may be because larvae or adults of this species consume more varied diets (therefore including more diverse bacterial species) than the other two species. Therefore, the higher level of bacterial richness could be associated with diets used; our study is thus similar to findings in previous studies showing that host diet seemed to affect the composition of gut microbiota in insects [42].

In this study we found *Ralstonia pickettii* as the only shared bacteria among all the three sand fly species in the steppe region. This species and other identified genera including *Bacillus*, *Enterobacter*, *Enterococcus*, *Staphylococcus* and *Ralstonia* are commonly related to plants common in the environments of *Phlebotomus* [26, 47]. Therefore, using plants visited by sand flies for sap consumption could be considered as a delivery method for introducing manipulated bacteria for a paratransgenesis approach [48].

Sand fly species of *P. major* and *P. kandelakii* are well known vectors of ZVL in Iran and other countries [10, 49–51] and *P. halepensis* is a susceptible vector of *L. infantum* in Georgia as well as *L. major* and *L. tropica* in other countries [52]. *Phlebotomus kandelakii* plays an important role in ZVL transmission in Iran and is suggested as the main vector of ZVL in both northeastern (steppe) and northwestern (mountainous) areas of the country [13]. Moreover, this species is a major vector of ZVL in other countries such as Georgia [53] and Turkey [54]. The high bacterial diversity in *P. kandelakii* could be linked to its vectorial capacity. A recent laboratory study on midgut microbiomes in *Lutzomyia longipalpis*, the major vector of VL in new world, suggests that the gut microbiota of the sand fly is an important factor for the replication and development of *Leishmania* spp. and parasite conversion to infective metacyclic promastigotes before transmission to a new host [55]. Furthermore, the study by Fraihi et al. [35] showed seasonal variations in microbiota composition of the midguts of female *P. perniciosus* with a species diversity decline to the end of the *L. infantum* transmission period. The influence of insect gut microbiota on vector capacity of mosquitoes and tsetse flies has also already been reported [56, 57]. Interestingly, in the present study, eight bacterial species (27.27%) were linked to the family *Enterobacteriaceae* and detected in both steppe and mountainous regions and *P. kandelakii* and *P. major*. Members of this family have been reported in medically important insects [22, 24, 28, 58, 59] and involved in dietary supplementation, tolerance to environmental perturbations and maintenance and/or enhancement of host immune system homeostasis [57, 59–62]. Further investigations are needed to clarify possible relationships between these bacteria and the sand fly vector capacity for ZVL [55].

The detection of *Bacillus subtilis* and *Pantoea agglomerans* in sand flies studied here, suggests that these two bacterial species are potential candidates for the prevention of *Leishmania* transmission *via* paratransgenesis approaches [26, 35, 63, 64]. *Pantoea agglomerans* and *B. subtilis* have been used for the production of paratransgenic mosquitoes [65] and paratransgenic sand flies, respectively [16].

In this study we used a culture dependent-method and subsequently, differences in gut microbiota could be dissimilar from these resulting from the use of a culture independent-method. Culturing can potentially exclude slower-growing bacteria and those incapable of propagating on the test media; therefore, the diversity of the midgut microbiota still remains incomplete. Culture-independent approaches such as NGS provide a broader and deeper picture of gut microbiota diversity in host organisms [66]. However, we still need to search for cultivable bacteria present in insects because culturing bacteria still offers the best way of observing the diverse characteristics of the isolated organisms; the physiological characteristics such as antibiotic resistance, interspecies growth inhibition or population dynamics within vector cohorts of bacterial isolates can be determined. Also culturing facilitates bacterial genome sequencing, a further link towards revealing functionality [67], and availability of the candidate bacteria allows testing their malleability to accept foreign DNA (genes or plasmids).

Some of the limitations of the present study include a lack of next generation sequencing (NGS) facilities and not taking into account the age, sex, parity, gonotrophic cycle, blood meal, leishmanial infection and abdominal situation (gravid, semi gravid, empty) of the samples.

## Conclusions

The present study has provided a detailed investigation of the composition and richness of the gut microbiota in three sand fly species from two ZVL foci with distinct geographical features using a culture-dependent method and sequencing of the *16S* rRNA gene. Furthermore, this study has shown differences between the taxonomic composition of bacteria that could be recovered in cultures from midguts of three different sand fly species isolated from steppe and mountainous regions. The study of sand fly microbiota is important due to the finding of given bacterial species on various sand fly species in different locations may lead to the development of paratransgenic approaches targeting multiple vectors in various localities to control the spread of leishmaniasis. Although there are a number of limitations, this study provides basic information on aerobic bacteria for a potential paratransgenesis strategy in the guts of insects and the associations of microbes and their hosts.

## Abbreviations

NTD: Neglected tropical disease
L: *Leishmania*
ZVL: Zoonotic visceral leshmaniasis
P: *Phlebotomus*
VL: Visceral leishmaniasis
NGS: Next-generation sequencing
BHI: Brain heart infusion
OTUs: Operational taxonomic unit
